# Functional and Structural Overview of G-Protein-Coupled Receptors Comprehensively Obtained from Genome Sequences

**DOI:** 10.3390/ph4040652

**Published:** 2011-04-13

**Authors:** Makiko Suwa, Minoru Sugihara, Yukiteru Ono

**Affiliations:** 1 Computational Biology Research Center (CBRC), National Institute of Advanced Industrial Science and Technology (AIST), 10th Floor, AIST Waterfront Annex Building, 2-4-7 Aomi, Kotou-ku, Tokyo 135-0064, Japan; E-Mails: sugihara-minoru@aist.go.jp (M.S.); yuki-ono@aist.go.jp (Y.O.); 2 Information and Mathematical Science Lab. Inc., Meikei Building, 1-5-21 Ootsuka, Bunkyou-ku, Tokyo 112-0012, Japan

**Keywords:** GPCR, SEVENS database, genome, gene structure, PDB

## Abstract

An understanding of the functional mechanisms of G-protein-coupled receptors (GPCRs) is very important for GPCR-related drug design. We have developed an integrated GPCR database (SEVENS http://sevens.cbrc.jp/) that includes 64,090 reliable GPCR genes comprehensively identified from 56 eukaryote genome sequences, and overviewed the sequences and structure spaces of the GPCRs. In vertebrates, the number of receptors for biological amines, peptides, *etc.* is conserved in most species, whereas the number of chemosensory receptors for odorant, pheromone, *etc*. significantly differs among species. The latter receptors tend to be single exon type or a few exon type and show a high ratio in the numbers of GPCRs, whereas some families, such as Class B and Class C receptors, have long lengths due to the presence of many exons. Statistical analyses of amino acid residues reveal that most of the conserved residues in Class A GPCRs are found in the cytoplasmic half regions of transmembrane (TM) helices, while residues characteristic to each subfamily found on the extracellular half regions. The 69 of Protein Data Bank (PDB) entries of complete or fragmentary structures could be mapped on the TM/loop regions of Class A GPCRs covering 14 subfamilies.

## 1. Introduction

G-protein-coupled receptors (GPCRs) are membrane proteins characterized by seven transmembrane (TM) helices. They respond to various ligands such as, biological amines, peptides, hormones, and odorant substances, from the extracellular side of the cell. Such stimulations induce GPCRs to activate G-proteins and to transmit signals to the interior of the cell. GPCRs exist in most cells and the abnormal signal transduction of GPCRs is related to various serious conditions, such as allergy, heart trouble, cancer, high blood pressure, and inflammation, *etc.* As approximately 30% of the medicines distributed worldwide are designed to control this receptor system [1,2], many researchers in both academia and industry are trying to reveal the functional mechanisms of GPCRs. For a long time, experimental difficulties in both structure determination and gene expression process had prevented researchers from understanding the functional mechanisms of GPCRs. However, recently, a large amount of genome information from many species has become available and several new structures of GPCRs have been revealed. In this context, it is now possible to overview sequences and structure spaces to understand the general rule of GPCR function by using bioinformatics. For this purpose, we have identified GPCR genes from the genome sequences of various species and have stored available information from approximately 60 subfamilies in an integrated database (SEVENS http://sevens.cbrc.jp/). From the viewpoint of collecting GPCR sequences, many useful GPCR databases are already available: GPCRDB [3], IUPHAR (GPCR database) [4], GPCR-PD™ [5], ORDB [6], gpDB [7], Human-gpDB [8], and GLIDA [9], *etc.* GPCRDB is the most popular database and includes known GPCR sequences from UniProt and GENBANK. IUPHAR and GPCR-PD™ accumulate bibliographic information as well as sequence information. ORDB focuses on the olfactory receptor, a subfamily of GPCRs. Furthermore, unique data regarding the interaction of a GPCR with G-proteins, and with effectors are summarized in gpDB and Human-gpDB, whereas the relationship between GPCRs and their ligands are included in GLIDA. These databases are well-organized and useful for the analysis of known GPCR genes. However, to perform an overview of all GPCR genes, it is necessary to treat a comprehensive dataset that should include not only the expressed sequences but also the newly identified sequences that cannot be detected by *in vivo* experiments, although they definitely exist on the genome sequence. The SEVENS database is suitable for this purpose. In this review, we report the results of a survey of sequence and structure information of GPCRs using the SEVENS database.

## 2. Method: SEVENS Database

First, we identified GPCR genes from genome sequences using a computational gene discovery pipeline that is composed of the GPCR gene finding stage and the GPCR gene screening stage [10-12]. At the gene finding stage, the genomic regions to which exon sequences of known GPCRs showed significant match were selected by using TBLASTN and complete gene structures were constructed from exon regions by using the ALN program [13]. Then, we defined a gene as a pseudogene when a stop codon was found at the exon region or when there was a frame shift by insertion or deletion. The most accurate dataset (96.6% sensitivity and 99.4% specificity) was obtained by combining several outputs that were calculated by using the optimized threshold of the sequence similarity search by BLASTP, the GPCR specific Pfam domain assignments by HMMER [14], and the TM helix prediction by SOSUI [15] at the gene screening stage. Finally, screened sequences that showed hits to known GPCRs in UniProt (http://www.uniprot.org/) with E-values < 10^−30^ against BLASTP search were categorized to the same subfamily of known GPCRs. Short protein sequences with less than 150 residues were eliminated.

**Figure 1:**
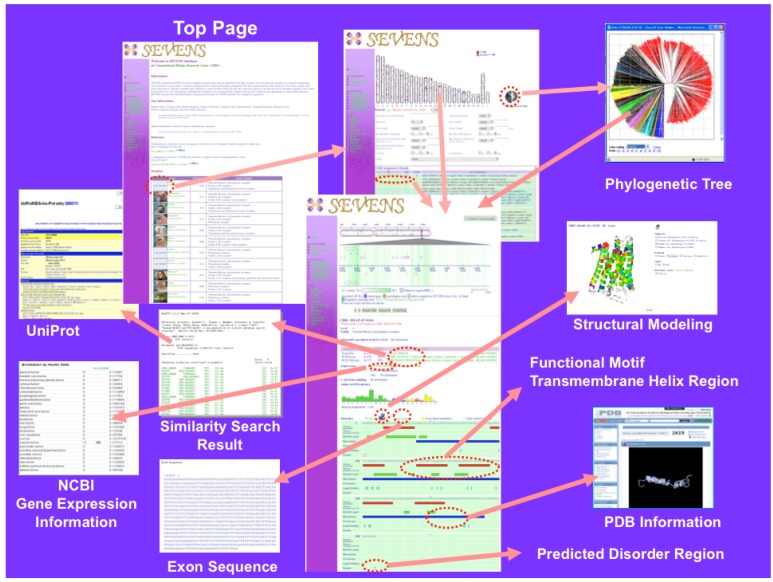
WEB presentation of SEVENS database. By selecting the species on the top page, one can access a new page with the search list, the phylogenetic tree viewer, or the chromosomal map. This new page is linked to the gene annotation information page in which structural and functional information is found, and one can access other information pages.

Currently, the SEVENS database (version 1.70) stores 64,090 genes from 56 eukaryotes and more than half (35,125 genes) of the total genes are olfactory receptor. It is an integrated database in which various kinds of functional and structural information of each GPCR gene are visually presented and organized in a hierarchical manner. Figure 1 shows the Web page of the SEVENS database. The top page shows a list of eukaryote genomes. When a species type is selected, the entry search page is shown. Users can access the gene annotation information page from the chromosomal map, the phylogenetic icon, or the search condition entry form.

In the gene annotation information page, the genetic coordinate of a selected GPCR is displayed on the chromosomal map together with information of known regulatory regions, GC contents, and other genes near the query. Furthermore, such information as sequence similarity search results, gene expression patterns, ligand binding, G-protein binding, amino acid sequence composition, predicted TM helix regions by SOSUI [15], functional motif regions, domain regions, predicted disorder regions by DISOPRED [16], exon-intron boundaries, pseudogenes, novel genes, and regions of known structures, is available.

Because several three-dimensional (3D) structures [17-25] had already been elucidated for the Class A (Family 1) type GPCRs, we could assign actual TM helix regions of this Class. The TM regions of the Class A GPCRs were determined based on the available crystal structures of bovine rhodopsin, adrenergic receptors, and adenosine receptors to which multiple sequence alignments of the selected sequences were assigned with the avoidance of gaps in the TM regions. Based on this information, the actual TM helix regions are displayed on the gene annotation information page, and a modeled structure of selected a Class A GPCR using the comparative modeling method is presented by Jmol viewer [26]. The sequence regions that show significant match to the large coverage region of the PDB sequence are represented by purple bars.

Using the aligned sequences we performed the statistical analysis of subfamily distribution, gene structure, amino acid residue conservation, and 3D structure information. For the analysis of subfamily distribution and gene structure, we used all sequences deposited in SEVENS database [10-12]. For the analysis of conserved residues in GPCRs, we retrieved the sequences of Class A GPCRs and selected 1,388 sequences from 49 vertebrate species in each subfamily, by comparing sequences in SEVENS with those in UniProt, with the criteria: Amino acid identity and sequence coverage rate are more than 80% and 90%, respectively. And finally, 1,272 sequences having complete lengths, which belong to 48 subfamilies of 31 vertebrate species, except olfactory/gustatory receptors, were considered for statistical analysis.

## 3. Results and Discussion

### 3.1. Annotated Subfamily Distribution

**Figure 2:**
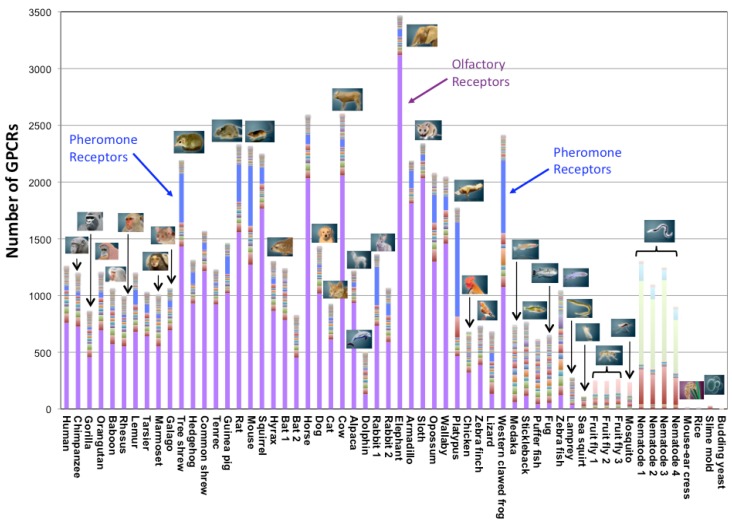
Number of GPCRs in 58 eukaryotes. Data from 56 eukaryotes (except sloth and horse) are deposited in SEVENS database. Each bar represents the total number of GPCRs with subfamily information indicated by different colors. Olfactory and pheromone receptors of vertebrates are in purple and blue, respectively. The specific names of members in four species are as follows. Bat1: *Pteropus vampyrus*; Bat2: *Myotis lucifugus*; Rabbit1: *Oryctolagus cuniculus*; Rabbit2: *O. princeps*; Fruit fly1: *Drosophila melanogaster*; Fruit fly 2: *D. simulans*; Fruit fly 3: *D. yakuba*; Nematode 1: *Caenonhabditis elegans*; Nematode 2: *C. remanei*; Nematode 3: *C. brenneri*; Nematode 4: *C. briggsae*.

In eukaryotes, we identified a few GPCRs in yeasts, a dozen in plants, approximately 200 in insects, several hundreds in fishes and birds, and from several hundreds to several thousands in mammals. When we broke down those numbers into the subfamily classification (Figure 2), the number of subfamilies (represented by different colors in Figure 2) was around several in the lower species. It increased rapidly in the higher species after the fish, and became nearly 60 in mammals (e.g., 57 subfamilies in the case of human). Within those subfamilies, the number of receptors for biological amines, peptides, lipids, *etc.* is conserved in mammals, birds, and fishes. Currently, eight subfamilies are commonly found in all species, except for Yeasts and Plants. They are acetylcholine receptors, adrenaline receptors, dopamine receptors, serotonin receptors, Class B (Family 2) receptors, Class C (Family 3) receptors, Frizzled/Smoothened receptors, and glycoprotein receptors. The first four receptors affect nervous system control and the remaining four are related to the interaction between cells. (Genes of yeasts and plants are excluded from this analysis because they have a small number of GPCRs, making it difficult to apply comparative genome analysis.)

On the other hand, the number of receptors for chemical substances is distributed uniquely in different species. For example, the percentage of olfactory receptors (colored in purple in Figure 2) is approximately 70% in GPCR genes and shows much diversity in mammals. The percentage of olfactory receptors is approximately 60% in human and primates, and is extremely high (more than 80%) in elephant, armadillo, sloth, cow, horse, squirrel, *etc.* In contrast, one sea animal (dolphin) has a rather small number of olfactory receptors. Other types of olfactory receptors that are peculiar to insects (Drosophila odorant receptor) account for approximately half of all the genes of two kinds of insects (Drosophila and mosquito). It is interesting that the number of pheromone receptors (colored in blue in Figure 2) increases only in certain species: Tree shrew, rabbit, mouse, rat, platypus, and western clawed frog. More than 700 nematode chemosensory receptors are found in C. elegans. In addition, around 130 trace amine receptors are found, particularly in zebrafish.

Interestingly, such chemical substance receptors that are characteristic to each species show biased distribution on the genome sequences. Those receptors form large clusters in which several dozens of genes are found at high densities. For example, large clusters appear on human chromosome 11, mouse chromosome 1, dog chromosomes 18 and 21, and C. elegans chromosome 5, *etc.* In contrast, genes found in most of the other subfamilies are sparsely distributed on the genome sequences. Previous studies have also suggested that species-specific families form gene clusters on the genome sequences [27-32]. In general, it is suggested that gene duplication occurs on the genome sequence when the number of cluster members increases.

From the viewpoint of comprehensive collection of GPCRs, Fredriksson *et al*. [33] created a phylogenetic tree of approximately 800 known human GPCR sequences and proposed a new taxonomy, “GRAFS,” which consisted of five main groups. This was applied to 13 kinds of eukaryotes [34]. With regards to the comprehensive GPCR gene analysis of individual species, many publications, including those that deal with insect [35], plant [36], human and mouse [37,38], and human and dog [39], are available. The GPCR gene number collected by our pipeline is in good agreement with the previous studies, although it is somewhat larger [10-12].

### 3.2. Gene Structure

**Figure 3:**
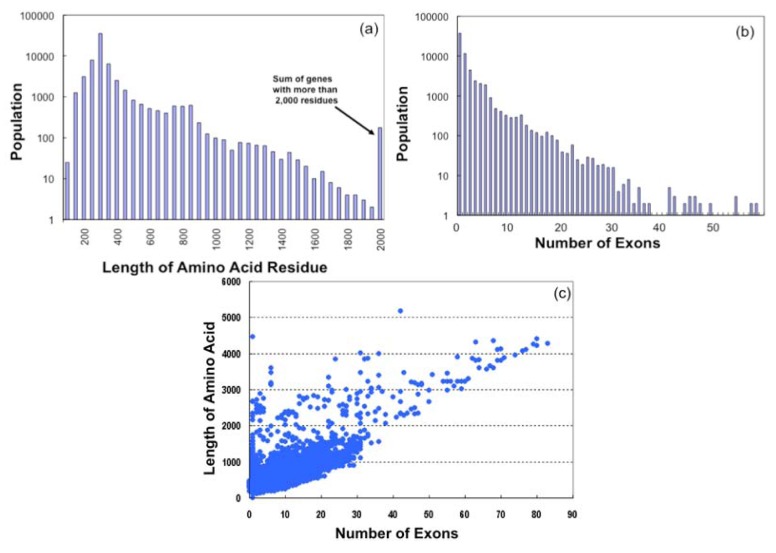
Statistics of gene structures. (**a**). Amino acid length distribution of GPCRs; (**b**). exon number distribution of GPCRs; (**c**). amino acid length (*L*) population plotted as a function of number of exons (*N*).

On examination of the amino acid lengths of GPCR sequences (Figure 3a), it was found that most of the GPCR sequences contain 250 ∼ 400 residues (367 residues on average), which are mainly the lengths of olfactory receptor sequences, while such receptors as Class B and Class C types have more than 1,000 residues. The longest receptor sequence was found in Class C, which has 5,179 amino acid residues.

Based on the exon-intron mapping information stored in the SEVENS database, we obtained the number of exons for each gene. Figure 3b suggests that most GPCR genes in 64,090 sequences are of the single exon type (37,459, ca. 58%) or a few exon type which are consisted of mostly olfactory receptors. In contrast, the number of genes with more than 10 exons is 2,465, with a Class C receptor gene having the largest exon number (*N*_max_ = 83). The large number of exons in most of such genes is due to the extracellular loop regions of Class B and Class C receptors. Figure 3c shows the amino acid length (*L*) plotted as a function of the number of exons (*N*). Large clusters are found in the region where *L* < 2000 and *N* < 30, and a linear relationship (*L* ∼50_*_N) is observed where *N* > 30.

From the gene mapping information, we observed that exons have long dispersion genomic regions of 2,000,000 nt at most. It is natural that GPCR genes with a large number of exons require long genomic regions, although there is no clear correlation between the number of exons and the length of the dispersion region of each exon. We found a gene with 14 exons requiring the longest dispersion region (1,982,894 nt) and this region is longer than that of the gene with the largest exon number (*N*_max_ = 83).

Based on our method of gene identification, the genomic coordinate of some GPCRs is the product of prediction and is not confirmed by experiments. However, the results of statistical analysis seem to be similar to that of previous work that dealt with all of the genes [40-42]. In contrast, GPCR-specific features are observed in our analyses, *i.e.*, the content of single-exon-type sequences mainly related to olfactory receptors is high.

### 3.3. Residue Conservation

To understand the functional mechanism of GPCRs, it is important to study the amino acid residues conserved in GPCR sequences. Conserved residues at the extracellular side affect ligand-binding selectivity, whereas those at the cytoplasmic side affect G-protein coupling selectivity. Many articles have reported the positions of conserved residues [43-49] and the mutation experiments of key residues that have significant influence on the ligand binding or G-protein coupling selectivity [43,45-47,49].

For statistical analysis of conserved residues, we retrieved the sequences of Class A GPCRs deposited in the SEVENS database [10-12], because it is the major Class that accounts for approximately 70% of all GPCRs and several full-length 3D structures for this families have already been revealed [17-25]. The 1,272 sequences, having complete lengths, from 48 subfamilies of 31 vertebrate species, except olfactory/gustatory receptors, were considered for statistical analysis (See “Method section” for detail). Here, strongly conserved residues in the 1,272 sequences were observed in each TM helix region: Asn (100%, *Asn55: residue with the corresponding position of bovine rhodopsin*) in TM1; Asp (97%, *Asp83*) in TM2; Cys (90%, *Cys110*), Ser (82%, *Ser*127), Asp (68%, *Glu134*), Arg (98%, *Arg135*), and Tyr (74%, *Tyr136*) of DRY motif in TM3; Trp (97%, *Trp161*) and Pro (63%, *Pro170*) in TM4; Pro (82%, *Pro215*) and Tyr (88%, *Tyr223*) in TM5; Phe (84%, *Phe261*), Cys (75%, *Cys264*), Trp (78%, *Trp265*) and Pro (100%, *Pro267*) of CWxP motif in TM6; and Asn (80%, *Asn302*), Pro (95%, *Pro303*), and Tyr (92%, *Tyr306*) of NPxxY motif in TM7. Most of these conserved residues are concentrated on the cytoplasmic side of TM helices while residues characteristic to each subfamily were found on the extracellular side. It is suggested that DRY motif area form hydrogen bonding networks between surrounding TM helices [48] and is important for G-protein coupling selectivity [49]. This motif area is almost fully occupied by a major combination of Asp-Arg-Tyr (57%) or Glu-Arg-Tyr (10%).

Statistics of the 1,272 vertebrate sequences show that the length of the third cytoplasmic loop (CL3) varies considerably (average: 34), ranging from 1 (chemokine receptor) to 230 (acetylcholine receptor). A similar tendency was observed in the N- and C-terminal loops. It is interesting that almost all loops of these N- and C-terminal loops and CL3 are predicted to have disorder regions by using DISOPRED program [16]. In contrast, the other loops have nearly the same lengths: 7 (the first Extracellular Loop (EL) 1), 24 (EL2), 13 (EL3), 7 (the Cytoplasmic Loop (CL) 1), and 12 (CL2) with several residues dispersion at most, suggesting that the structures around TM 1, 2, 3, and 4 are similar.

### 3.4. Structure Space

Because GPCRs exist in the membrane environment, crystallization is difficult and the number of 3D structures available is very small. However, in the past two to three years, the number of 3D structures of GPCRs has increased rapidly. β_1_- and β_2_-adrenergic receptors [18-20], A_2A_ adenosine receptors [21], and squid rhodopsin [22,23] were crystallized and their 3D structures completely solved. Furthermore, in 2010, the structures of CXCR4 chemokine [24] and D3 dopamine receptors [25] were successfully determined. This situation depends greatly on recent technological developments [50], such as (1) a method to express large amounts of functioning receptors by introducing genes into insect cell membrane; (2) technology for crystallization promotion by CL3 modification due to insertion of a globular protein or binding to an antibody protein; (3) crystallization technology in the environment that is very similar to that of native membrane; and (4) data extraction technology from a fine crystal with a very bright X-ray beam-line having less than 10 μm width.

Beside the PDB entries of complete structures (29 structures of five subfamilies), there are many fragmentary structures related to GPCRs. For Class A GPCRs, we can compute the statistics of the fragmentary structures mapped on the TM helix and loop regions as the actual boundary of the two regions can be obtained by alignment to known structures [17-25].

**Figure 4:**
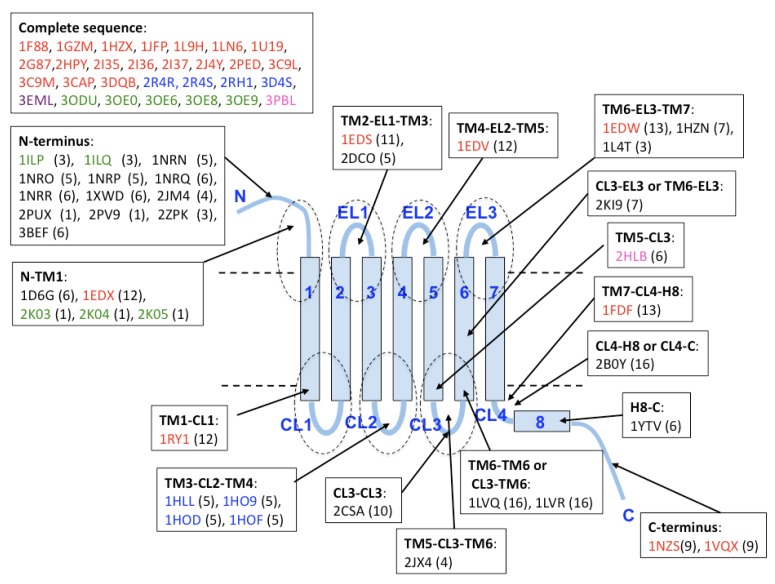
Schematic representation of PDB data that are annotated to Class A type entry sequences in SEVENS database. The 3D structures of five subfamilies were solved completely: bovine rhodopsin (colored in red), adrenergic receptor (in blue), adenosine receptor (in magenta), chemokine receptor (in green), and dopamine receptor (in pink). Fragments of these receptors are shown in the same color code. Number in parenthesis represents the hit count of PDB regions to the sequences identified in SEVENS database.

These positions are schematically shown in Figure 4. For fragmentary structure mapping, we used the criterion of more than 80% similarity between whole sequences in PDB and those in the SEVENS database (see Methods section) and therefore, one PDB structure matches several sequences in SEVENS. The 69 PDB entries in Figure 4 are the products of X-ray crystallography (38 structures), NMR measurement (30 structures) or electron microscopy (1 structure), although the coordinates predicted by comparative modeling are excluded. We observed 22 fragmentary structure entries on the N- and C-terminal loop regions (18 in N-terminus and 4 in C-terminus) and 18 in the other loops, which included few residues of the edge of TM regions. The resolution of those structures varies from 1.2 Å (the most accurate resolution) to 4 Å. The structure of bovine rhodopsin shows the largest number (26 entries), because it can be determined in many experimental conditions. These coordinates cover 14 subfamilies (muscarinic receptor, acetylcholine receptor, adenosine/adenine nucleotide receptors, adrenergic receptor, cannabinoid receptor, chemokine/chemotactic factor receptors, cholecystokinin/gastrin receptor, dopamine receptor, glycoprotein hormone receptor, lysolipid receptor, opsins, proteinase-activated receptor, relaxin receptor, and vasopressin receptor).

Meanwhile, most of the revealed structures for Class B and Class C GPCRs are the products from long N- and C-terminal loops. It should be carefully examined why some of the domains of non GPCR proteins (globular proteins), such as tubulin-like domain, SH3-like domain, *etc.*, showed a significant match with long extracellular loops of GPCR families (bile acid receptor, trace amine receptor, somatostatin and urotensin receptors, olfactory and gustatory receptors, melanocortin receptor, oxytocin receptor, and UNKNOWN types). Given the current situation that 3D structures are still rare for GPCRs, this is important information, even though they are only fragmentary structures.

## 4. Conclusions

In this review, we overviewed the sequences and structure spaces of GPCRs in the integrated SEVENS database from the viewpoint of family distribution, gene structure, amino acid residue conservation, and 3D structure. As the dataset stored in SEVENS has been identified accurately and comprehensively from complete genome sequences, the available information and the results of analyses are expected to reveal the GPCR proteome and the evolutional diversity of GPCR functions.

It is necessary to take into account the recent achievements in the research on the 3D structures of GPCRs. For a long time, bovine rhodopsin is the only GPCR whose 3D structure has been solved and it has been used as a template for the comparative modeling of GPCRs analyzed in drug discovery researches. However, from 2007 to 2010, the GPCR structures of different families [17-25] were determined by X-ray crystallography and conventional research methods are expected to change rapidly.

From the new 3D structures, the diversities in the structures of the ligand binding sites and the G-protein binding sites have become clear and should be considered in the case of studying different families. Thus, it is necessary to determine the 3D structures of all major GPCR families. However, such high throughput structure determination is impossible because the producing functional receptors in the membrane and crystallization of receptors are bottlenecks. Therefore, structural information must be obtained through a new approach that does not directly depend on 3D structure determination. It is important to extract and overview information that reflects the available 3D structures for each family at the sequence level by bioinformatics, and the SEVENS database has been constructed to serve this purpose.
